# The Role of Multidisciplinary Ocular and Periocular Cancers Meetings in Uveal Melanoma Management: A 2-Year Analysis

**DOI:** 10.3390/cancers17142274

**Published:** 2025-07-08

**Authors:** Gustavo Savino, Monica Maria Pagliara, Maria Grazia Sammarco, Carmela Grazia Caputo, Maria Antonietta Blasi, Roberta Mattei, Sofia Marcelli, Luca Tagliaferri, Bruno Fionda, Giovanni Schinzari, Ernesto Rossi, Luca Zagaria, Tommaso Tartaglione, Luca Ausili Cefaro, Mattia Todaro, Alessandro Moro, Federico Giannuzzi

**Affiliations:** 1 Ocular Oncology Unit, Fondazione Policlinico Universitario A. Gemelli IRCCS, 00168 Rome, Italy; gustavo.savino@unicatt.it (G.S.); monicamaria.pagliara@policlinicogemelli.it (M.M.P.); mariagrazia.sammarco@policlinicogemelli.it (M.G.S.); carmelagrazia.caputo@policlinicogemelli.it (C.G.C.); mariaantonietta.blasi@unicatt.it (M.A.B.); roberta.mattei@policlinicogemelli.it (R.M.); sofia.marcelli@guest.policlinicogemelli.it (S.M.); 2 Ophthalmology Department, Università Cattolica del Sacro Cuore (UCSC), 00168 Rome, Italy; 3 UOC Radioterapia Oncologica, Dipartimento di Diagnostica per Immagini, Radioterapia Oncologica ed Ematologia, Fondazione Policlinico Universitario A. Gemelli IRCCS, 00168 Rome, Italy; luca.tagliaferri@policlinicogemelli.it (L.T.); bruno.fionda@policlinicogemelli.it (B.F.); 4 Medical Oncology Unit, Fondazione Policlinico Universitario A. Gemelli IRCCS, 00168 Rome, Italy; giovanni.schinzari@policlinicogemelli.it (G.S.); ernesto.rossi@policlinicogemelli.it (E.R.); 5 Nuclear Medicine Unit, Fondazione Policlinico Universitario A. Gemelli IRCCS, 00168 Rome, Italy; luca.zagaria@policlinicogemelli.it; 6 Radiology Department, Fondazione Policlinico Agostino Gemelli IRCCS Hospital, 8 Largo Agostino Gemelli, 00168 Rome, Italy; tommaso.tartaglione@policlinicogemelli.it (T.T.); luca.ausilicefaro@policlinicogemelli.it (L.A.C.); 7 Maxillo-Facial Surgery Unit, IRCSS Fondazione Policlinico Universitario “A. Gemelli”-Università Cattolica del Sacro Cuore sede di Roma, 8 Largo Agostino Gemelli, 00168 Rome, Italy; mattia.todaro@policlinicogemelli.it (M.T.); alessandro.moro@policlinicogemelli.it (A.M.); 8 Ophthalmology Unit, Fondazione Policlinico Universitario A. Gemelli IRCCS, 00168 Rome, Italy

**Keywords:** uveal melanoma, ocular oncology, ocular oncology multidisciplinary tumor board, multidisciplinary team, ocular MDTB

## Abstract

**Purpose**: The objective of this study was to evaluate the implementation of a Multidisciplinary Tumor Board (MDTB) strategy in the treatment of patients with uveal melanoma. **Methods**: A retrospective analysis was conducted on the implementation of MDTB meetings over a 24-month period. During this time, 72 intraocular tumors were discussed, including 59 confirmed cases of uveal melanoma. The MDTB involved a core group of specialists (e.g., ophthalmologists, oncologists, and radiologists), with other experts included when clinically appropriate. To assess patient satisfaction with the MDTB approach, a structured questionnaire was administered, including items on clarity of communication, perceived quality of care, and overall satisfaction, which were ranked on a 5-point scale. **Results**: A total of 319 patients with ocular, periocular, or orbital tumors were discussed during the study period, of which, 72 had intraocular tumors. A total of 13 (18%) were diagnosed to have choroidal metastases, whereas 59 (82%) had uveal melanomas. The average time between patient care and MDTB discussion was 15.9 days (IQR: 7.5–16.5). The mean time between the case discussion and the implementation of recommendations (diagnostic, therapeutic, or referral decisions) was 14.8 days (IQR: 6.0–23.75). Overall, 4 (7%) patients were classified as Stage I, 16 (27%) as Stage IIa, 18 (31%) as Stage IIb, 7 (12%) as Stage IIIa, 2 (3%) as Stage IIIb, and 12 (20%) as Stage IV. Regarding the satisfaction questionnaire, all patients (100%) agreed to have the clinical case discussed at the TB even though this could result in a delay in diagnostic/therapeutic implementation. However, only 60% of patients perceived they had been directly involved in the decision-making process. **Conclusions**: In selected cases of uveal melanoma and other types of cancer, MDTBs should be recognized as a gold standard in cancer care, allowing for comprehensive decision-making that draws on a wide range of highly specialized expertise.

## Simple Summary

This research assessed the efficacy of a Multidisciplinary Tumor Board (MDTB) approach in the management of patients with uveal melanoma. In a span of two years, 319 cases of ocular tumors were analyzed, comprising 59 patients diagnosed with uveal melanoma. The mean duration from patient referral to MDTB discussion was approximately 16 days, with treatment decisions executed within 15 days. The majority of patients received diagnoses at intermediate or advanced stages. All patients expressed support for having their cases reviewed by the MDTB, despite the potential for a brief delay in treatment. Only 60% reported active involvement in decision-making. The study demonstrates that MDTBs enhance treatment planning through the inclusion of specialists from various fields and should be implemented as a standard practice in the management of complex cancers such as uveal melanoma.

## 1. Introduction

Uveal melanoma (UM) represents the most prevalent primary intraocular malignancy in adults, with a mean incidence of 5.1 cases per million each year [1,2]. UM treatment primarily aims to eradicate the tumor, to prevent recurrences and metastasis, and, whenever feasible, to preserve visual function [3].

Classification, staging, and treatment modalities should, however, comply with the latest national and international guidelines [4,5,6,7].

Significant progress has been achieved in recent years in terms of risk stratification assessment, types of treatment, follow-up methods, and the handling of metastatic forms [4,5,6,7].

Genetic profiling together with clinical features and staging according to AJCC 8th ed. staging is crucial for a proper risk profiling, and the several options for eye sparing treatment (brachytherapy, fractionated stereotactic radiotherapy, and proton beam radiation) require the availability of advanced devices and trained and dedicated staff (geneticists, molecular biologists, radiotherapists, and nuclear physicists) [6,8,9].

To date, enucleation is limited to cases with neovascular glaucoma, blind and painful eyes, tumors replacing more than 50% of the globe and involving the optic disc, and tumors with extraocular extension [10,11].

The heterogeneity of the disease, the availability of multimodal treatments, and the promising new systemic adjuvant and neo-adjuvant treatments that have been approved or are in an advanced trial phase require a multi-specialist approach [12].

Multidisciplinary Tumor Board (MDTB) meetings are increasingly recognized as a gold standard in cancer care, allowing for comprehensive decision-making that draws on diverse expertise [13,14].

The MDTB has been shown to enhance adherence to clinical guidelines in the treatment of a variety of malignancies when it is integrated into cancer care [15,16,17].

MDTs primarily operate through regular meetings that focus on the discussion of clinical cases involving cancer patients, resulting in tailored diagnoses and treatment plans for each patient [18]. Each meeting includes new cases and follow-up discussions.

The objective of this study was to evaluate the implementation of a Multidisciplinary Tumor Board (MDTB) strategy in the treatment of patients with uveal melanoma. A Multi-Disciplinary Tumor Board (MDTB) for Ocular Oncology was established at our tertiary referral center in recent years. Our study aimed to analyze the team composition, the main UM discussed, features, topics, and decisions made.

## 2. Materials and Methods

The authors analyze the activity of the MDTB of the Ocular Oncology Unit at “Fondazione Policlinico Universitario A. Gemelli IRCSS” in Rome from January 2023 to November 2024. The study was conducted in accordance with the Declaration of Helsinki. The study was approved by the Catholic University/Fondazione Policlinico Universitario A. Gemelli IRCCS Institutional Ethics Committee (protocol ID number: 5703). A signed informed consent was obtained from each enrolled patient. Once every two weeks, meetings were held at our tertiary referral center and a retrospective analysis of each presented cases was carried out, followed by a final report with the findings and decisions (diagnostics, request for referrals, and treatments). Cases of ocular surface, periocular, orbital, and intraocular tumors were discussed within the meetings. In this study, however, only the cases of uveal melanomas were included. Most of the uveal melanoma cases were treated according to international guidelines and not discussed at the MDTB [10].

The following are the cases that came to the attention of the tumor board: suspected extra-scleral extension, uncertain diagnosis and nature, suspected distant metastasis, and cases requiring a multidisciplinary opinion; clinical and/or histopathological diagnosis, staging or restaging, and treatment planning were discussed.

The following data were collected: (a) main site involved; (b) main points discussed during meeting; and (c) final outcomes (clinical decision and recommendations).

All patients diagnosed with UM underwent a comprehensive ocular examination.

The assessment included best-corrected visual acuity, slit lamp biomicroscopy with imaging, fundus photography, gonioscopy, ultrasonography, and indirect ophthalmoscopy.

An ultrasound examination was conducted utilizing a high-frequency transducer for the assessment of iris and ciliary body cancers, alongside B-scan and A-scan techniques for posterior tumors. Tumor thickness was assessed at the thickest point, and longitudinal and transverse dimensions were recorded. In the case of iris and ciliary body melanomas, we systematically assessed the entire 360° span of the anterior segment, in addition to evaluating tumor elevations and diameters. Additional high-frequency ultrasound features were assessed, including tumor morphology, scleral infiltration, internal vascularization, and tumor reflectivity. MRI images were also available for all cases discussed.

All of the cases are uploaded to an IT platform, Healthmeeting, which facilitates the storage of data and photos while enabling real-time reporting of team findings.

The follow-up of patients varied based on the clinical presentation and severity of the condition.

### 2.1. MDTB Composition

As previously reported, the board consisted of a “core team” of ocular oncologists, medical oncologists, radiation oncologists, radiologists, pathologists, maxillofacial surgeons, and oto-laryngologists [12]. Furthermore, a care manager, a data manager, and an MDTB meeting coordinator were also incorporated. The core team members consistently attended meetings, while an extended team of experts, including dermatologists, nuclear physicians, hematologists, and abdominal surgeons, participated when required [12].

### 2.2. Satisfaction Questionnaire

A short questionnaire was administered to the patients subsequent to the MDTB decision, which including the following questions:

1. Would you be in favor of having your case discussed in a multidisciplinary meeting? Meetings are held fortnightly and this could lengthen the clinical-therapeutic decision time. Response: yes or no.

Patients who answered “Yes” proceeded to the following questions, each scored on a 5-point Likert scale (1 = not at all, 5 = very much):

2. Discussing your case in a multidisciplinary meeting how much more confident you feel in the suggested treatment? Answer score range 1 to 5.
3. How involved do you feel in the decision-making process about your case? answer score range 1 to 5.
4. Would you like to be involved in multidisciplinary meetings whenever possible? Answer score range 1 to 5.
5. Do you believe that a multidisciplinary discussion can provide a stronger adherence of the suggested clinical-therapeutic pathway to the international guidelines? answer score range 1 to 5.

This questionnaire was not derived from a previously validated instrument but was specifically developed for this study to explore patient perceptions of the MDTB approach. Although not formally validated, its content was reviewed by clinicians and a small group of patients to ensure clarity and relevance. The 5-point scale was chosen to allow gradation of the responses and facilitate descriptive statistical analysis.

## 3. Results

From January 2023 to November 2024, 72 cases of intraocular tumors out of a total of 319 ocular or periocular cancers treated at our ocular oncology unit were discussed. A total of 85 (27%) were diagnosed as ocular superficial tumors, 72 (23%) as intraocular tumors, 70 (22%) as orbital tumors, 82 (25%) as adnexal tumors, and 10 (3%) were undiagnosed cases. Among the 72 intraocular tumors, 13 (18%) were diagnosed as choroidal metastases and 59 (82%) as uveal melanomas. During the 23-month study period, a total of 265 uveal melanoma were first observed at our ocular oncology unit, 22.2% (59 cases) of which were screened for discussion.

Among them, 49 cases were posterior choroidal melanomas; in 7 cases, the tumor involved the ciliary body and choroid, and in 2 cases, the ciliary body and iris were involved.

Five cases were suspected of extra-scleral growth. In all these cases, the images were reviewed together with the neuroradiologist during the discussion.

Approximately 20% of the discussed cases were in the metastatic disease stage. In a further 5%, metastatic lesions were suspected, and further imaging was discussed or the patient was referred for further examination (i.e., liver biopsy).

Metastatic patients in IV stage (12 patients, 20%) were discussed to define the proper diagnostic–therapeutic approach: further imaging (1 case), hepatectomy (1 case), radiosurgery (2 cases), systemic therapy (6 cases), radiotherapy (1 case), and enucleation (1 case).

The median diameter of the lesions was 4.35 mm (IQR: 3.48-7.52). The median thickness was 15.07 mm (IQR: 12.75–17.00).

**Figure 1:**
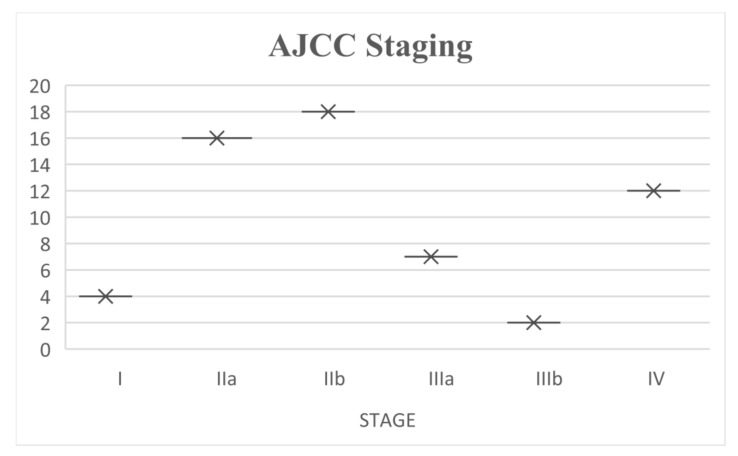
AJCC 8th staging at presentation of 59 patients with uveal melanoma.

The patients were categorized according to AJCC 8th ed, as showed in Figure 1. Overall, 4 (7%) patients were Stage I, 16 (27%) patients were stage IIa, 18 (31%) patients were stage IIb, 7 (12%) patients were stage IIIa, 2 (3%) patients were stage IIIb, and 12 (20%) patients were stage IV.

**Figure 2:**
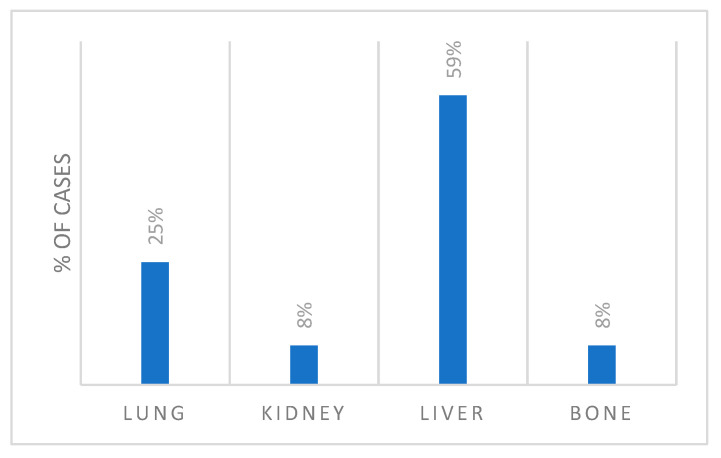
Metastasis distribution at presentation.

Seven cases (59%) in stage IV had liver metastasis, three (25%) had lung metastases, one patient (8%) had bone metastasis, and one patient (8%) had kidney metastasis (Figure 2).

A total of 5 cases, other than the definitive 59, were initially diagnosed as UM and presented for discussion. Multidisciplinary discussion of the clinical and imaging features changed the diagnoses to metastatic choroidal tumors.

The mean time between patient caretaking and MDTB discussion was 15.9 days (IQR: 7.5–16.5).

The mean time between patient discussion and recommendation (diagnostic, therapeutic, or referral decision) implementation was 14.8 days (IQR: 6.0–23.75). The decisions provided by the MDTB are showed in Table 1.

Regarding the patient satisfaction questionnaire, all patients agreed to have their clinical case discussed at the TB even though this could result in a delay in implementing therapeutic treatment. For the second query (Discussing your case in a multidisciplinary meeting how much more confident you feel in the suggested treatment?), 70% of the patients gave a score of 5 and 30% gave a score of 4.

For the question, How do you feel involved in the decision-making process about your case? only 35 (60%) patients gave a score of 5, 11(18%) gave a score of 4, 9 (15%) gave a score of 3, 4 (3%) gave a score of 2, and only 1 (1%) gave a score of 1.

Regarding their willingness to be involved in multidisciplinary meetings whenever possible, all the patients (100%) reported that they were willing to be involved in the discussion. With regard to the last query (Do you believe that a multidisciplinary discussion can provide a stronger adherence of the suggested clinical therapeutic pathway to the international guidelines?), 41 (70%) patients gave a score of 5, 12 (20%) gave a score of 4, 6 (10%) responded with “I don’t have enough knowledge to answer”.

The responses are reported in Table 2.

## 4. Discussion

MDTB discussions represent the benchmark in cancer care, facilitating thorough decision-making through the integration of varied expertise. The integration of the MDTB into oncology has shown enhanced adherence to clinical protocols when treating various malignancies, with the literature primarily indicating improved survival outcomes in several cancer types [12,18]. Patients can find reassurance in the fact that the treatment recommendations align with clinical guidelines, leading to improved standardization and quality of care. Additionally, MDTB sessions offer educational benefits to participants. This platform facilitates knowledge sharing among professionals, benefiting junior staff in training and promoting lifelong learning among specialists. The participants are informed about emerging treatments and proposed clinical trials. However, there is limited evidence concerning the application of this approach in ocular oncology.

Shah et al. reviewed the data collected over a 12-month period. The meetings were effective in defining a shared management of patients and had a positive psychological effect on the patients [19].

Savino et al. reported that imaging (MRI or CT scans) evaluations were the main topic in the discussions of orbital tumors, while histopathologic reports were often analyzed in eyelid tumor cases [12].

Discussions about intraocular tumors mainly concerned treatment planning through multimodal approaches (enucleation, orbital exenteration, or brachytherapy) for particular cases (large tumors, extrascleral suspected extension, and suspected metastasis).

In this study, we analyzed the usefulness and limitations of multidisciplinary meetings for UM cases over a 23-month period.

The total number of cases (265) at our center and the number of cases discussed (59) in this time period is certainly significant even in view of the low incidence of this cancer in Europe and in Italy, with <2 cases per million persons in Spain and southern Italy and >8 per million in Norway and Denmark [20].

Despite effective management of the primary lesion, 40–50% of patients with UM ultimately develop distant metastases, most commonly involving the liver (93%), lungs (24%), bones (16%), and/or soft tissues (11%) [21].

Owing to the limited efficacy of available regional and systemic therapies, until recently, the historical median overall survival (OS) of these patient following clinical detection of metastasis was around 1 year [22,23].

Recently several international collaborations have clarified the processes underlying UM risk stratification, development, spreading, dormancy, and progression. A number of promising treatment strategies (neoadjuvant and adjuvant) are now being evaluated in several clinical trials.

In January 2022, tebentafusp, a bispecific T cell-engager targeting CD3 and glycoprotein 100 (gp100) in an HLA-A*02:01-restricted, has been approved for the treatment of metastatic UM after an overall survival benefit was demonstrated in an international phase III trial [24].

This advance gives hope for significant treatment improvements for this disease. Nevertheless, this also requires an increasingly multispecialty and integrated approach to the disease, and multidisciplinary discussions become particularly crucial in defining uncertain diagnoses and managing complex cases with suspected or confirmed metastases.

In our study, the patients with typical presentations were treated according to standard protocols, but a significant percentage of the observed cases of UM (22.2%) were discussed.

In view of the new therapeutic possibilities and numerous clinical trials, it has become crucial to discuss these cases with abdominal imaging experts, liver surgeons, radiation oncologists, and medical oncologists, preferably at a highly specialized referral center, although this could lead to a mild lag in the implementation of the treatment plan.

One study reported that multidisciplinary discussion rarely (5.2%) modifies the clinical diagnosis [19]. In our sample, the MDTB discussions changed the initial diagnosis of uveal melanoma into choroidal metastasis in five cases (2% of all the cases examined and 7% of intraocular tumors evaluated). Both cases had a low average thickness and amelanotic tumors, and MRI examinations revealed minimal melanin levels in T2-weighted scans. Further investigations (total body CT scan, mammography, and thyroid ultrasound) led to amending the diagnosis as lung and breast carcinoma metastases.

All patients, while advised of a likely delay in the diagnostic–therapeutic decision, agreed to have their case discussed. Yet, only 60% felt involved in the decision-making process. Improvement could be achieved via telephone or email correspondence with the patient during the interim period preceding the MDTB decision or by engaging them through video conversations during the clinical case presentation. All patients would appreciate a greater involvement in the discussion of their case. Most (70%) of patients perceived that the TB case discussion provided a higher degree of adherence to the national and international guidelines.

This study has multiple limitations. The absence of a control group complicates the comparison of outcomes between MDTB-managed and unmanaged cases. Consequently, it remains unclear whether the MDTB improved the therapeutic efficacy or patient outcomes. This study utilized an unvalidated patient satisfaction questionnaire. The absence of psychometric validation (including reliability testing and construct validity) may restrict its interpretability and generalizability. This study did not evaluate cost-effectiveness. MDTBs have the potential to enhance care quality and clinical decision-making; however, their long-term effects on healthcare costs, resource allocation, and workflow efficiency are still unclear. Further investigations are needed to analyze the costs, time, and resources needed for MDT meetings; they require significant time and logistical coordination, which can be challenging in busy healthcare settings, especially if there is a shortage of specialists.

## 5. Conclusions

With the availability of new treatments, even for advanced and metastatic UM, more accurate risk stratification and staging are needed, which require an ever more multi-specialist approach and the need for continuous learning and updating processes.

International guidelines must often be implemented considering the specific features of the clinical cases, the challenge of some diagnostic referrals, and the need to involve dedicated medical staff to achieve a supported team decision. To enhance MDTBs, it is essential to implement strategies that promote increased patient involvement, including soliciting pre-meeting input and gathering post-meeting feedback. Telemedicine could be used to enhance specialist involvement and minimize delays. Subsequent investigations should evaluate the survival outcomes and cost-effectiveness, and validate the measures reported by the patients. It is essential to conduct comparative studies that include control groups. The standardization of MDTB processes will enhance reproducibility and impact. 

## Figures and Tables

**Table 1:** Decisions provided by MDTB for patients affected by uveal melanoma.

MDTB Outcome	n = 59
Referral to Liver Surgeon	2 (4%)
Ruthenium Plaque Brachytherapy	12 (21%)
Liver Radiotherapy	2 (4%)
Ocular Oncology Follow-Up	7 (12%)
Further Imaging Request	12(21%)
Enucleation	4 (7%)
External Beam Radiotherapy	1(1%)
Referral to Medical Oncologist	12 (20%)
Proton Beam Therapy	5 (9%)
Referral to Radiotherapist	1 (1%)
Transscleral Resection	1(1%)

**Table 2:** Responses to questionnaire.

Question	1 (n=)	2 (n=)	3 (n=)	4 (n=)	5 (n=)	No Score
Discussing your case in a multidisciplinary meeting how much more confident you feel in the suggested treatment?	-	-	-	17	42	-
How involved do you feel in the decision-making process about your case?	1	4	9	11	35	-
Would you like to be involved in multidisciplinary meetings whenever possible?	-	-	-	-	59	-
Do you believe that a multidisciplinary discussion can provide a stronger adherence of the suggested clinical-therapeutic pathway to the international guidelines?	-	-	-	12	41	6

## Data Availability

Data are available from the corresponding author, F.G., upon reasonable request.

## References

[1] Singh A.D., Turell M.E., Topham A.K. (2011). Uveal melanoma: Trends in incidence, treatment, and survival. Ophthalmology.

[2] Krantz B.A., Dave N., Komatsubara K.M., Marr B.P., Carvajal R.D. (2017). Uveal melanoma: Epidemiology, etiology, and treatment of primary disease. Clin. Ophthalmol..

[3] Damato B., Heimann H. (2013). Personalized treatment of uveal melanoma. Eye.

[4] Diener-West M., Earle J.D., Fine S.L., Hawkins B.S., Moy C.S., Reynolds S.M., Schachat A.P., Straatsma B.R. (2001). The COMS randomized trial of iodine 125 brachytherapy for choroidal melanoma, III: Initial mortality findings. COMS Report No. 18. Arch. Ophthalmol..

[5] Dell’Istituto Superiore di Sanità (2024). Diagnosi e Trattamento del Melanoma Uveale.

[6] Hawkins B.S. (2004). The Collaborative Ocular Melanoma Study (COMS) randomized trial of pre-enucleation radiation of large choroidal melanoma: IV. Ten-year mortality findings and prognostic factors. COMS report number 24. Am. J. Ophthalmol..

[7] Amin M.B., Greene F.L., Edge S.B., Compton C.C., Gershenwald J.E., Brookland R.K., Meyer L., Gress D.M., Byrd D.R., Winchester D.P. (2017). The Eighth Edition AJCC Cancer Staging Manual: Continuing to build a bridge from a population-based to a more “personalized” approach to cancer staging. CA Cancer J. Clin..

[8] Rao P.K., Barker C., Coit D.G., Joseph R.W., Materin M., Rengan R., Sosman J., Thompson J.A., Albertini M.R., Boland G. (2020). NCCN Guidelines Insights: Uveal Melanoma, Version 1.2019. J. Natl. Compr. Cancer Netw..

[9] Hussain R.N., Chiu A., Pittam B., Taktak A., Damato B.E., Kacperek A., Errington D., Cauchi P., Chadha V., Connolly J. (2023). Proton beam radiotherapy for choroidal and ciliary body melanoma in the UK-national audit of referral patterns of 1084 cases. Eye.

[10] NCCN Guidelines-Melanoma: Uveal.

[11] McLean L.C.I.W., Foster W.D., Zimmerman L.E. (1982). Uveal melanoma: Location, size, cell type, and enucleation as risk factors in metastasis. Human. Pathol..

[12] Savino G., Piccinni F., Pagliara M.M., Sammarco M.G., Caputo C.G., Moro A., Barbera G., Tagliaferri L., Fionda B., Schinzari G. (2022). Multidisciplinary ocular and periocular cancers meetings: Implementation in a tertiary referral center and analysis over a 12-months period. BMC Ophthalmol..

[13] El Saghir N.S., Keating N.L., Carlson R.W., Khoury K.E., Fallowfield L. (2014). Tumor boards: Optimizing the structure and improving efficiency of multidisciplinary management of patients with cancer worldwide. American Society of Clinical Oncology Educational Book.

[14] Keating N.L., Landrum M.B., Lamont E.B., Bozeman S.R., Shulman L.N., McNeil B.J. (2013). Tumor boards and the quality of cancer care. J. Natl. Cancer Inst..

[15] Vinod S.K., Sidhom M.A., Delaney G.P. (2010). Do multidisciplinary meetings follow guideline-based care?. J. Oncol. Pract..

[16] Krause A., Stocker G., Gockel I., Seehofer D., Hoffmeister A., Bläker H., Denecke T., Kluge R., Lordick F., Knödler M. (2023). Guideline adherence and implementation of tumor board therapy recommendations for patients with gastrointestinal cancer. J. Cancer Res. Clin. Oncol..

[17] Walter J., Moeller C., Resuli B., Kauffmann-Guerrero D., Manapov F., Dinkel J., Neumann J., Kovacs J., Schneider C., Huber R.M. (2023). Guideline adherence of tumor board recommendations in lung cancer and transfer into clinical practice. J. Cancer Res. Clin. Oncol..

[18] Licitra L., Keilholz U., Tahara M., Lin J.-C., Chomette P., Ceruse P., Harrington K., Mesia R. (2016). Evaluation of the benefit and use of multidisciplinary teams in the treatment of head and neck cancer. Oral. Oncol..

[19] Shah M.A., Lynch E., Cauchi P., Chadha V. (2019). One Year of the Ocular Oncology Multidisciplinary Team Meeting—Has it Made a Difference?. Clin. Oncol. (R. Coll. Radiol.).

[20] Virgili G., Gatta G., Ciccolallo L., Capocaccia R., Biggeri A., Crocetti E., Lutz J.M., Paci E. (2007). Incidence of uveal melanoma in Europe. Ophthalmology.

[21] Collaborative Ocular Melanoma Study Group (1998). Histopathologic characteristics of uveal melanomas in eyes enucleated from the Collaborative Ocular Melanoma Study. COMS report no. 6. Am. J. Ophthalmol..

[22] Khoja L., Atenafu E.G., Suciu S., Leyvraz S., Sato T., Marshall E., Keilholz U., Zimmer L., Patel S.P., Piperno-Neumann S. (2019). Meta-analysis in metastatic uveal melanoma to determine progression free and overall survival benchmarks: An international rare cancers initiative (IRCI) ocular melanoma study. Ann. Oncol..

[23] Rantala E.S., Hernberg M., Kivelä T.T. (2019). Overall survival after treatment for metastatic uveal melanoma: A systematic review and meta-analysis. Melanoma Res..

[24] Carvajal R.D., Nathan P., Sacco J.J., Orloff M., Hernandez-Aya L.F., Yang J., Luke J.J., Butler M.O., Stanhope S., Collins L. (2022). Phase I Study of Safety, Tolerability, and Efficacy of Tebentafusp Using a Step-Up Dosing Regimen and Expansion in Patients with Metastatic Uveal Melanoma. J. Clin. Oncol..

